# NAD^+^ deficit, protein acetylation and muscle aging

**DOI:** 10.18632/aging.203177

**Published:** 2021-06-07

**Authors:** Li Li Ji, Dongwook Yeo

**Affiliations:** 1 Laboratory of Physiological Hygiene and Exercise Science, School of Kinesiology, University of Minnesota Twin Cities, Minneapolis, MN 55455, USA; 2 Department of Orthopedic Surgery, Mayo Clinic, Rochester, MN 55905, USA

**Keywords:** aging, SIRT, NAD ^+^, deacetylation, skeletal muscle

Despite decades of research, the cellular mechanism of age-related muscle loss and functional deterioration (sarcopenia) is still unclear. Increased protein acetylation plays an important role in mitochondrial and functional decline in aged muscle [1]. Proteomic analysis revealed that over 100 lysine sites in mitochondrial proteins may be acetylated, which could be one of the most common post-translational modification of mitochondrial homeostasis [2]. Increased acetylation and inactivation of PGC-1α decreases mitochondrial biogenesis and function, while acetylation of key enzymes in the TCA cycle and ETC leads to declined oxidative phosphorylation [3]. Acetylation of SOD2 suppresses it catalytic activity and elevates superoxide anion levels in the mitochondrion leading to oxidative damage to lipids, protein and mitochondrial DNA. Furthermore, acetylation of p65 subunit of NFκB facilitates its entry into the nucleus and increases DNA binding, a crucial step in the transactivation of pro-inflammatory cytokine expression [4]. Thus, maintenance of cellular protein acetylation/deacetylation balance is critical for cell survival during aging.

Protein deacetylation is carried out mainly by the NAD^+^-dependent deacetylase SIRTs, during which the acetyl groups from lysines are transferred to NAD^+^, forming NAM and O-acetyl-ADPr [3]. Previous studies suggest that aging downregulates SIRT1 activity thus undermining cellular function and increasing inflammation; however, SIRT1 protein levels show no decline with age [4]. Some studies even reported positive correlations between SIRT content and age in muscle (see Yeo et al. [5]). In these studies, SIRT protein was examined in various tissues from different animal species, therefore whether aging upregulates or downregulates SIRT expression in skeletal muscle remains unclear. The study by Yeo et al. [5] showed that 24-month old mice had much higher protein levels of SIRT1,3,5,6 in two muscle groups and cardiac muscle, whereas GCN5 level was unaltered or even decreased. However, aged muscles still displayed elevated acetylation of several key enzymes (SOD2, GCN5) and transcription factor/cofactors (PGC-1α, p65), as well as total protein content. Thus, cellular SIRT levels do not seem to determine the extent of protein acetylation/deacetylation status, at least in skeletal muscle. Importantly, the authors showed that NAD^+^ contents in both muscles, and in both cytosol and nucleus, were markedly decreased at old age. This finding raised a possibility that with aging, the insufficient supply of NAD^+^ may be a limiting factor in intracellular deacetylation capacity despite increased SIRT expression.

**Figure 1:**
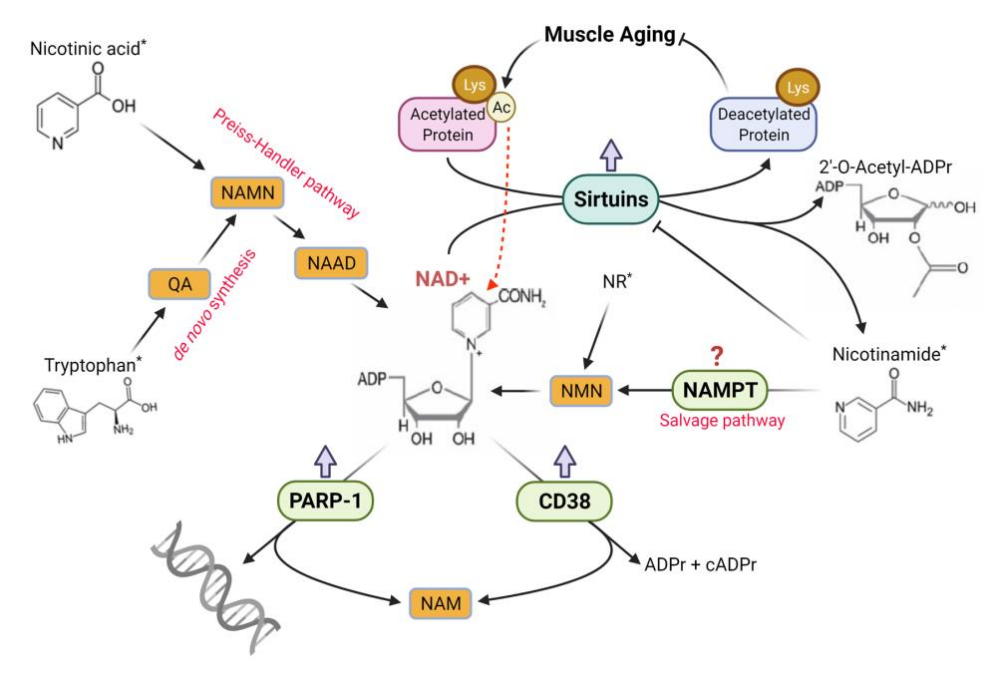
**Schematic illustration of the regulation of NAD^+^ homeostasis in skeletal muscle during aging.** Arrows with blunt ends indicate inhibition. Red thick arrows denote direction of age-related changes. Compounds with an * denote dietary sources. Figure created with BioRender.com.

The question thus arises as to why aging diminishes cellular NAD^+^ pool. It is well established that metabolic pathways that use NAD^+^ and NADH as coenzyme to transfer electrons do not contribute to net NAD ^+^ loss in the cell, whereas NAD^+^ is primarily consumed by SIRTs, PARP-1, and CD38-catalyzed pathways [3,4]. During aging, each of the three major NAD^+^-consuming pathways may be activated and competes for NAD^+^ as a substrate (Figure 1). First, aging is a prominent inducer of mitochondrial protein hyperacetylation in muscle, which requires greater amount of NAD^+^ to accept acetyl groups from enzymes in TCA cycle, ETC and SOD2 [1]. These reactions are catalyzed by SIRT3, 4 and 5 [3]. Age-related activation of NFκB and FoxO pathways also demand NAD^+^ for SIRT1, 2, 5, 6, 7-catalyzed deacetylation in the nucleus. Second, PARP-1 uses NAD^+^ as a substrate to transfer pADPr backbone to repair damaged DNA base pairs. In fact, requirement for DNA repair is recognized to contribute to 80% of NAD^+^ decline, and aging is a known cause of DNA oxidative damage [3]. In skeletal muscles of old mice, PARP-1 was upregulated while cleaved PARP-1 and pADPr levels were also elevated [5]. Interestingly, PARP-1 inhibition was shown to improve mitochondrial function due to SIRT1 activation, presumably by increased NAD^+^ availability [6]. Third, aging increases CD38 protein level and NADase activity, whereas inhibition of CD38 or CD38 gene knockout was shown to rescue intracellular NAD^+^ and preserve SIRTs activity [7]. Consistent with this notion, CD38 protein content revealed 5-12 fold increases in aged muscles [5].

Theoretically, increased NAD^+^ consumption can be replenished by elevated NAD^+^ synthesis in the cell. The Preiss-Handler pathway converts dietary NA to NAMN, then NAAD, and finally NAD^+^, whereas the *de novo* NAD^+^ biosynthesis from dietary tryptophan requires multiples steps limited by TDO and IDO [3]. Whether the enzyme activities in the Preiss-Handler pathway and *de novo* biosynthesis pathway are downregulated or upregulated during aging is unclear. A more important pathway relied on by organisms is the NAD^+^ salvage pathway controlled by NAMPT, the rate-limiting enzyme converting NAM generated by SIRTs, CD38 and PARP-1 to NMN and eventually NAD^+^ (Figure 1). This pathway not only recycles NAM to maintain intracellular NAD^+^ level, but also relieves NAM inhibition on SIRTs [3]. There is some evidence that age-related decline of NAMPT activity could reduce NAM recycling and NAD^+^ salvage resulting in muscle degeneration [8]. However, Yeo et al. [5] demonstrated that NAMPT expression was upregulated in aged muscles, possibly as a compensatory mechanism. Recent research shows that dietary supplementation of NAM, or NR as a precursor of NAM, can boost intracellular and mitochondrial NAD^+^ levels, improve metabolic function and even increase longevity in aged mice [4].

In summary, available literature suggests that aged muscles suffer from NAD^+^ deficit due to increased competitions from SIRTs, CD38 and PARP-1 pathways. Whether the NAMPT-catalyzed NAD^+^ salvage pathway is upregulated or downregulated with aging is somewhat controversial, but clearly it is not sufficient for NAD^+^ recycling. Thus, despite upregulation of SIRT protein levels in aged muscle, *in vivo* SIRT activity can be severely hampered due to diminished NAD^+^, resulting in acetylation of key enzymes and transcription factors causing mitochondrial dysfunction and redox disturbance. These studies emphasized the importance of preserving intracellular NAD^+^ homeostasis and provided a mechanistic basis for research targeting new drug and/or dietary intervention for sarcopenia.
